# A holistic comparison of flavor signature and chemical profile in different harvesting periods of *Chrysanthemum morifolium Ramat.* based on metabolomics combined with bioinformatics and molecular docking strategy†

**DOI:** 10.1039/d2ra05698d

**Published:** 2022-12-07

**Authors:** Mengxin Yang, Xi Tian, Miaoting Zhang, Jinhuan Wei, Yukun Niu, Jiali Hou, Yiran Jin, Yingfeng Du

**Affiliations:** Department of Pharmaceutical Analysis, School of Pharmacy, Hebei Medical University Shijiazhuang Hebei 050017 P. R. China yingfengdu@hotmail.com +86-311-86266419 +86-311-86265625; The Second Hospital of Hebei Medical University Shijiazhuang Hebei 050000 P. R. China jinyiran@sohu.com +86-311-86266419 +86-311-86265625

## Abstract

Taiju and Duoju are products of Hangbaiju (HJ) obtained during different collection periods, and they have been commonly used as ingredients in tea beverages and dietary traditional Chinese medicine. This study reports an integrated strategy based on metabolomics, bioinformatics and molecular docking to further explore the effect of the harvesting period on the metabolic profile and clinical efficacy of HJ. Firstly, gas chromatography-mass spectrometry (GC-MS) and ultra-high performance liquid chromatography-quadrupole time-of-flight mass spectrometry (UHPLC-Q-TOF/MS) were employed for non-targeted metabolomics profiling of essential oils and flavonoids. A sequential window acquisition of all theoretical fragment-ion spectra information-dependent acquisition (SWATH-IDA) bi-directionally verified (SIBDV) method was developed that integrates the advantages of both SWATH and IDA in characterizing flavonoids. Chemometric methods were then used to screen potential chemical markers. Furthermore, HJ is effective in hepatoprotective functions. Therefore, hepatocellular-carcinoma-related differentially expressed genes were obtained using bioinformatics, and the corresponding proteins were molecularly docked with diagnostic chemical markers. In total, 78 volatile oils and 63 flavonoids were tentatively identified. The results allowed the selection of 11 metabolites (5 volatile oils and 6 flavonoids), which are nominated as novel markers for material authentication of Taiju and Duoju. Additionally, two proteins associated with hepatoma were screened using bioinformatics. All six flavonoid markers with binding energies of <−5 kcal mol^−1^ were considered to be anti-hepatoma biomarkers. Noticeably, in Taiju, the content of hydroxygenkwanin showed a downward trend, but the content of the other five flavonoids and the five flavored volatile difference compounds had an upward trend. This bestows a unique flavor profile on Taiju, leading to differences in sensory aroma and clinical efficacy in Taiju and Duoju. In conclusion, the transformation of secondary metabolites was the dominant trend during HJ growth. These findings lay the foundation for food development and distinguishing clinical applications.

## Abstract 2

This paper analyzed the differences between the two harvesting periods (Taiju and Duoju) on the ingredients and pharmacological activity in *Chrysanthemum morifolium Ramat.*

## Graphical abstract

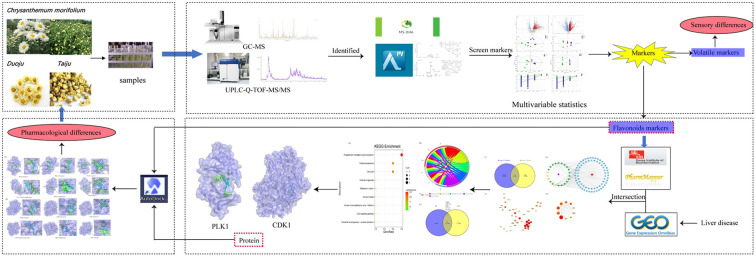

## 1 Introduction

The flowers of *Chrysanthemum morifolium* Ramat., *Flos Chrysanthemi*, which are cultivated in Zhejiang Province as ‘Hangbaiju’ (HJ), have been popular for 2000 years in China as a medicinal and edible cognate.^1^ The nutritional value of HJ was described and affirmed as early as the *Compendium of Materia Medica*. HJ is rich in many non-volatile and volatile metabolites that play a substantial role in flavor quality and health functions.^2^ Various volatile compounds determine the quality of tea aroma; these are composed mostly of diverse volatile compounds in different concentrations.^3^ There are many non-volatile metabolites, such as flavonoids, amino acids, phenolic acid compounds, *etc.* Among these, flavonoids have been shown to make a crucial contribution to the anti-inflammatory and hepatoprotective health functions of HJ.^4^ It is recorded in the *Chinese Pharmacopoeia* that *Chrysanthemum* has the effect of clearing the liver and brightening the eyes. Thus, it was used to prevent and treat liver damage.^5^ It is well-documented that *Chrysanthemum* extract significantly inhibits the proliferation of HepG2 and MHCC97H cells.^6,7^ These hepatoprotective effects of *Chrysanthemum* extract are probably mediated through antioxidative and antiapoptotic pathways.^8^ HJ, which is grown in the cities of Tongxiang and Jiaxing in the province of Zhejiang, is a cultivar of the Asteraceae family, and is listed as a national geographical indication of product protection.^9^ There are two main products of HJ, Taiju and Duoju, which are harvested at different times. The sensory differences between Taiju and Duoju have been demonstrated through previous research.^10^ The research showed that the content of volatile oil was positively correlated with the e-nose response value.^11,12^ These two types of HJ have unique flavor characteristics, which are closely related to their metabolite composition. In recent years, research on HJ has mainly focused on the qualitative and quantification of several unique components.^13,14^ However, there is a poor understanding of the differences between products from different harvesting periods, and the harvesting period of *Chrysanthemum* is not marked in herbal formulas. Hence, there is an impetus to establish a complete quality control technology platform to characterize and distinguish the two medicinal materials.

Recent research has revealed that gas chromatography-mass spectrometry (GC-MS) and ultra-high performance liquid chromatography-quadrupole time-of-flight mass spectrometry (UHPLC-Q-TOF/MS) based untargeted metabolomics approaches combined with multivariate analysis can be used to assess food quality in an objective and reliable manner.^15–17^ Currently, plant metabolomics has the capacity for the comprehensive analysis of herbal compounds, involving the screening of high-throughput compositional data. It could reveal the subtle differences in the kinds of ingredients or contents of ingredients between different plants and find the material basis of metabolic variation.^18,19^ The combination of UPLC, GC and MS can provide comprehensive information for the qualitative analysis of components.^20^ Multivariate statistical analysis has also been used to study the statistical regularity of the interdependence of multiple variables in objective things.^21,22^

Chemometric analysis plays an essential role within the metabolomics research process, as it provides a comprehensive understanding of large data sets in food science and technology.^23,24^ Chemometric techniques, mainly including principal component analysis (PCA) and orthogonal projection to latent structure discriminant analysis (OPLS-DA),^25^ are frequently used to deal with this type of data. Additionally, the bioinformatics methodology based on the Gene Expression Omnibus (GEO) database is capable of screening out differential genes from the massive transcriptomics data.^26^ At the same time, interactions between ligands and receptor active sites can be predicted with the aid of energy-based scoring functions in molecular docking methods, whereby the pharmacological activity of food components can be evaluated.^27,28^ In short, this is not only a powerful strategy for detecting multiple classes of components as well as screening for differential metabolites but also provides ideas for evaluating bioactive substances.

The accurate mass-to-charge ratios (*m*/*z*) recorded in the MS and MS/MS spectra play a critical role in the structure determination of UHPLC-Q-TOF MS samples. Information-dependent acquisition (IDA) and sequential windowed acquisition of all theoretical fragment ions (SWATH) are two distinct methods for selecting precursor ions.^29^ Traditional IDA methods are typically used to select precursors and acquire MS2 masses, and IDA can theoretically provide a high-quality MS2 spectrum, but requires a longer cycle time and complex settings. Although longer exclusion times help prevent repetitive trigger events for a compound, they may also lead to the loss of active ingredients and reduce MS2 hit rates.^30^ Recently, a new data-independent acquisition method, variable window acquisition of SWATH mode, has been reported, which can continuously acquire the MS2 spectrum of the entire precursor ions.^31^ Before the TOF analysis is performed, precursor ions are selected with a variable Q1 mass window width. Compared to the traditional IDA-MS strategy, SWATH could significantly improve the hit rate of low-level and trace metabolites.^32^

**Fig. 1:**
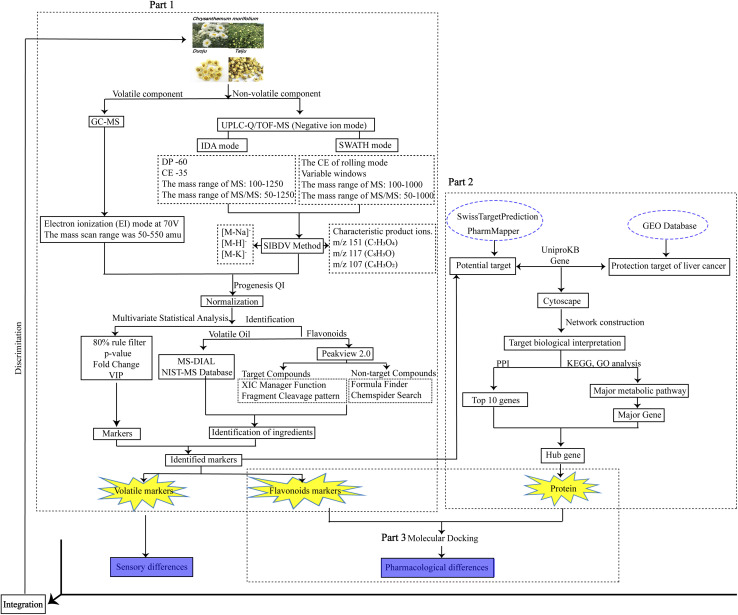
Workflow of the proposed approach for identifying and distinguishing HJ.

This study aimed to establish a high-throughput screening platform for the classification and identification of Taiju and Duoju through non-target metabolomics based on GC-MS and UHPLC-Q-TOF/MS combined with the chemometrics method. A comprehensive SWATH-IDA bi-directionally verified (SIBDV) method that incorporates the advantages of SWATH along with those of traditional IDA was then used to analyze large amounts of flavonoids in the two herbs. Finally, we analyzed the distribution of volatile oils and flavonoids using OPLS-DA with a heat map visualization. It was possible to clarify the differences and find chemical markers that could distinguish between all Taiju and Duoju batches using the technology platform. In addition, bioinformatics approaches based on the GEO database focused on genes with the greatest differences in expression between the liver cancer disease group and the normal group, which were combined with molecular docking techniques to screen for potential bioactive substances and to find biomarkers that distinguish the clinical efficacy of Taiju and Duoju. This approach is very promising in the development and design of functional HJ foods. The overall workflow chart is shown in Fig. 1.

## 2 Materials and methods

### 2.1 Chemical reagents and materials

HPLC-grade methanol and ethyl acetate were purchased from Tedia (Fairfield, USA). Purified water was supplied by Wahaha Corporation (Hangzhou, China). HPLC-grade formic acid was obtained from Fisher (Fair Lawn, NJ, USA). Anhydrous sodium sulfate (Analytical-grade, Tianjinyongda Chemical Reagent co., Ltd, P. R. China) was used for sample preparation. C7–C30 *n*-alkanes were purchased from Sigma-Aldrich (number: 49451-U) with 1000 μg mL^−1^ of each component in *n*-alkanes. Vitexin (170120), schaftoside (140402), diosmetin (101216), astragalin (101072), kaempferide-7-*O*-glucoside (20111603), hesperidin (20030407), quercitrin (20031704), quercimeritrin (20113007) and acacetin (20061901) were purchased from the SichuanWeikeqi Biological Technology Co., Ltd (Sichuan, China). Apigenin (ZL10852QCS), isoquercitrin (ZL54745YHP), luteolin (ZL10432MXC) and naringenin (ZL78156YPS) were provided by the Nanjing ZeLang medical technology co., Ltd (Nanjing, China). Baicalin (9SB3-UY4N), isorhamnetin (110860–200406), kaempferol (110861–200405), quercetin (100081–200406) and rutin (100080–200306) were obtained from the National Institute for the Control of Drugs and Biological Products (Beijing, China). Apigenin-7-*O*-glucuronide (BL18B013) was supplied by the Chengdu Push Bio-technology Co., Ltd (Chengdu, China). The purity of each reference component was no less than 98%.

The origin of all plant materials including Taiju and Duoju (28 batches, respectively) was Dongfeng *Chrysanthemum* Factory in Tongxiang, Zhejiang Province of China. They were authenticated by Professor Jianhua Wang at Hebei Medical University and stored in a desiccator at room temperature. A voucher specimen was deposited in the herbarium of the College of Pharmacy, Hebei Medical University.

### 2.2 Preparation of samples

A total of 28 batches were powdered to a homogeneous size and sieved through a no. 60 mesh sieve. Twelve of the batches with known classification were used for compositional identification and to search for differential metabolites, and the grouping of the other 16 batches were disrupted to the unknown in advance and then performed to validate the established differentiation model. Then, 30 mL of distilled water was added to 2 g of dry sample. Subsequently, the samples were extracted using a clevenger-type apparatus by hydro-distillation for 3 hours at room temperature. After dissolving the volatile oil in ethyl acetate, anhydrous sodium sulfate was used to dehydrate it. The supernatant was then filtered through a 0.22 μm membrane before being injected for GC-MS analysis. Furthermore, 2 g of dry sample was suspended in 10 mL of 50% MeOH/H_2_O solution, soaked for 1 h, and extracted with ultrasonication for 0.5 h at room temperature. Then, 50% methanol was added to compensate for weight loss at the end. The sample solution was centrifuged at 12 000 rpm for 10 min after transferring the supernatant to a 15 mL EP tube. Finally, the supernatant was filtered through a 0.22 μm filter membrane before injection for UHPLC analysis. Additionally, 19 separate stock solutions were prepared by dissolving an appropriate amount of each standard in 50% methanol. Afterward, the final mixed standard solution was prepared by mixing 50 μL of each stock solution. An equal volume of all samples analyzed was mixed for the preparation of pooled quality control (QC) samples to ensure broad metabolite coverage. It was injected throughout the analytical runs to further monitor the stability of the instrument status.

### 2.3 GC-MS and UHPLC-Q-TOF/MS analysis

The GC-MS system consisted of an Agilent 7890B GC system and 5977A series mass spectrometer with triple quadrupole (Agilent Technologies, USA). Chromatographic separation was conducted on a DB-17MS capillary column (30 m × 0.25 mm × 0.15 μm). The operating conditions of the GC system were as follows. Helium (99.999%) was the carrier gas with a flow rate of 1 mL min^−1^ throughout the analysis. The injection volume and split ratio were 5 μL and 10 : 1, respectively. Injection syringes were immediately washed with 5 μL ethyl acetate before and after each injection. The GC column temperature program was as follows: initially, 50 °C for 2 min, then a gradual increase to 130 °C at a rate of 5 °C min^−1^, and subsequently, rapid heating at 10 °C min^−1^ from 130 to 250 °C. For the mass spectrometry detection conditions, an electron ionization (EI) source with an ionization energy setting of 70 eV was used in this study. Temperatures of 260, 230, 150, and 280 °C were set for the injector, ion source, GC/MS interface, and quadrupole, respectively. In the full-scan acquisition mode, the mass range was 50–550 amu with a solvent delay of 3 minutes.

The LC-MS detection was carried out using a SHIMADZU LC-30A system coupled with a hybrid quadrupole time-of-flight tandem mass spectrometer with an electrospray ionization (ESI) interface (AB SCIEX, Triple-TOF 5600+, Foster City, CA, USA). Analytes were separated on a Phenomenex-C18 column (150 mm × 2 mm, 3 μm, Santa Clara, CA, USA) at 40 °C. The mobile phase consisted of 0.1% formic acid in water (A) and acetonitrile (B). The elution gradient was set as follows: 0–3 min, 5–10% B; 3–4 min, 25% B; 4–15 min, 95% B; 15–16 min, an isocratic elution of 95% B with equilibrium for 5 min. The flow rate and injection volumes were 0.3 mL min^−1^ and 2 μL, respectively. A pre-equilibration of 10 minutes followed to allow the instrument to equilibrate.

The mass spectrometry systems were both operated in negative ion electrospray mode. In IDA mode, the optimal operating conditions of MS were as follows: turbo spray temperature of 550 °C, ion spray voltage of −4500 V, declustering potential (DP) of −60 V, collision energy (CE) of −35 eV and collision energy spread (CES) of 15 eV for MS/MS. In this experiment, nitrogen was used as the nebulizer gas (GAS1), heater gas (GAS2) and curtain gas, which were set at 55, 55 and 35 psi, respectively. For the full MS scanning, the mass range was 100–1250 *m*/*z*. For production scanning, the range was from 50–1250 *m*/*z* with 0.2 s accumulation time. A total of eight ion precursors were selected from each full MS scan for MS/MS fragmentation, and dynamic background subtraction (DBS) was switched on. In addition, an automated calibration delivery system (CDS) was used every two hours to maintain mass accuracy. In SWATH mode, the scan range of precursor ion *m*/*z* was set as 100–1000 Da in full scan mode. The distributions of precursor ions were then used to calculate the SWATH MS acquisition windows using SWATH Variable Window Calculator_V1.0. There were twenty SWATH quadrupole isolation windows ranging from 50 to 1000 Da. The SWATH variable windows are shown in Table S1.† The CE voltage setting of the rolling mode was adopted to ensure high qualitative efficiency for flavonoids.

### 2.4 Summary of fragmentation patterns from standard compounds

Every standard solution and mixed standard was independently injected into the Triple TOF 5600+ mass spectrometer equipment, and the characteristics of the fragment ions and cleavage laws were determined. The fragmentation patterns of 19 standards were deduced from the TOF MS scan, Q1 scan, product ion scan and precursor ion scan for characterization. The molecular formula and molecular weight of the compound can be attained from the typical solvent adduct ions [M − Na]^−^ and [M − K]^−^ and protonated ion [M − H]^−^.

### 2.5 Metabolomics analysis

#### 2.5.1 Strategy of data processing

ABF converter was used to convert the raw data in the GC-MS results to “abf” format (http://www.reifycs.com/AbfConverter/index.html). Next, the converted data were pre-processed using MS-DIAL 4.70 (NSF-JST, Japan), including peak extraction, peak alignment, baseline calibration, deconvolution analysis and peak identification.^33,34^ We selected KovatsRI based on alkanes as a retention index for peak alignment. The deconvoluted spectra in NIST MSP format (GC-MS DB-Public-KovatsRI-VS3) were imported and matched with spectral libraries. Those peaks with an average peak width of 20 scans and minimum peak height above 10 000 amplitudes were selected for peak detection. Peaks with a σ-window value of 0.5 and EI spectral cutoff of 5000 amplitudes underwent a deconvolution operation. The identification parameters were set as follows: *m*/*z* tolerance of 0.5 Da, retention time tolerance of 0.5 min, EI similarity cutoff value of 70% and identification score cutoff value of 70%. The alignment parameters of retention time tolerance and retention time factor were set to 0.075 min and 0.5, respectively.

Raw data from UHPLC-Q/TOF-MS were processed using Progenesis QI software (Waters, USA) for peak detection, alignment and normalization. Flavonoids were identified as follows. The analytic strategy can be roughly classified into three parts. Firstly, a self-constructed database was constructed to assist in the characterization, including compound name, exact molecular weight and molecular formula.^35–38^ The fragmentation rules were summarized by analyzing the fragmentation behavior of 19 standards. Secondly, SIBDV was used to determine flavonoid profiles. First, IDA survey scans were initially used to measure chemical components. This was then able to provide a reference for the parameter settings of SWATH mode, obtain the SWATH spectral ion library and calculate the variable window. After that, SWATH was used to search for relevant ions in the low-concentration range, and then more comprehensive flavonoid ion information based on different CE and Q1 isolation windows was obtained. Thirdly, it was combined with the analysis of target compounds and non-target compounds to identify probable compounds. Target compounds were detected using XIC Manager and IDA Explore in PeakView 2.1, which matched mass spectral fragmentation rules of sample chromatograms with features from the in-house chemical library for analysis. The errors of the secondary fragmentation ions were set to 10 ppm. Among them, comparison of the retention times, secondary characteristic fragment ions and exact molecular masses of reference substances can accurately identify the components. Additionally, for no-target compounds, PeakView 2.0 software was employed to speculate potential structures using the search functions of Formula finder and ChemSpider.

#### 2.5.2 Multivariate statistical analysis

To further comprehensively evaluate quality and screen for differential markers between Taiju and Duoju, SIMCA-p 14.0 software (Umetrics, Uppsala, Sweden) was used for multivariate data analysis. Principal component analysis (PCA), an unsupervised multivariate statistical method, is used to view grouping trends and is widely used in the preliminary analysis of MS data sets. In addition, the stability of the instrument is reflected by the degree of aggregation of the QC samples in PCA. Orthogonal partial least squares discriminant analysis (OPLS-DA), a supervised discriminant analysis with a prediction function, can screen out variables that differ between groups. The coefficients of determination (R2Y, Q2Y) and the 200 times permutation test were also used to assess the model quality. In the OPLS-DA, the value of variable importance in projection (VIP) was used to screen the variables that would contribute to group discrimination. The components satisfying *p*-value < 0.05, fold change FC > 1.2 or FC < 0.8, and VIP > 1 in GC-MS or VIP > 3 in UPLC-Q/TOF-MS were considered significantly different metabolites. Finally, based on the MetaboAnalyst 5.0 online software (https://www.metaboanalyst.ca/), a heat map was generated to visualize the distribution of the potential markers, and the receiver operating characteristic (ROC) curve was applied to verify the accuracy of the potential diagnostic markers.

### 2.6 Bioinformatics analysis

In this study, the bioinformatics method was used to analyze differential genes of liver cancer, and the targets of flavonoids were searched for in databases. The predicted targets of the flavonoids were obtained from the following databases: PharmMapper (https://www.lilab-ecust.cn/pharmmapper/) and SwissTargetPrediction (https://www.swisstargetprediction.ch/). The GeneChip of the GSE84402 data set matrix data was downloaded from the GEO public database. Next, the VLOOOKUP function in Excel converted the gene symbols in the annotated probe matrix into gene annotation IDs. The samples of GSE84402 were grouped according to the data for liver cancer and non-cancerous tissues, and then the control group and experimental group were distinguished using Excel. Differential genes were obtained, and volcanoes were plotted using the limma package in R software according to the conditions |log FC| > 1 and *P* value < 0.005. The genes up-regulated in the experimental group are indicated in red, and the down-regulated genes in green. Subsequently, co-expressed genes were plotted using a Venn diagram. String online software was used to construct PPI network diagrams with a minimum interaction score of >0.400. Afterward, the PPI network was visualized using Cytoscape and the TOP10 genes were obtained using the Cytohubba plugin. Subsequently, hepatocarcinoma genes were screened out using ggplot2 graphs of GO and KEGG bubble maps. Finally, the stereoscopic structures of the core targets were obtained from the RCSB Protein Data Bank (http://www.pdb.org/).

### 2.7 Molecular docking

To explore the biological activity, the identified markers in the extracts were studied using a molecular modeling approach against liver cancer. The phytocompound ligands were used for molecular docking with the target protein for liver cancer using AutoDockTools-1.5.7 software. In the analysis, the protonation state of the protein was determined by adding hydrogen atoms to the protein structure without considering water molecules. Calculations for this study were conducted using the Lamarckian genetic algorithm.^39^ Docking effects were evaluated based on binding energies and action sites.

## 3 Results and discussion

### 3.1 The advantages of the SIBDV online data acquisition method in qualitative studies of TCM

**Fig. 2:**
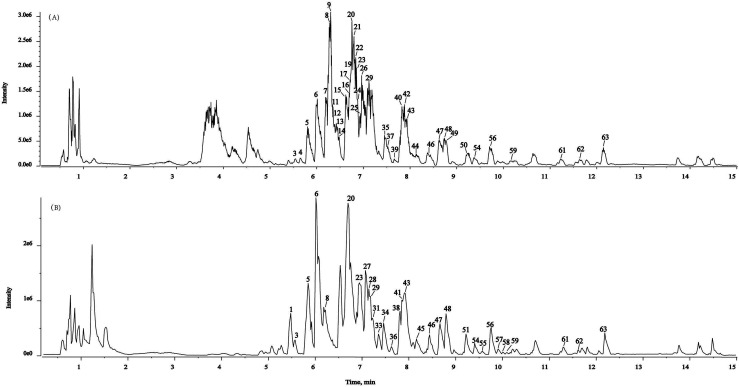
Representative base peak chromatogram (BPC) of Taiju samples acquired in IDA mode and SWATH mode. (A) IDA mode. (B) SWATH mode.

**Fig. 3:**
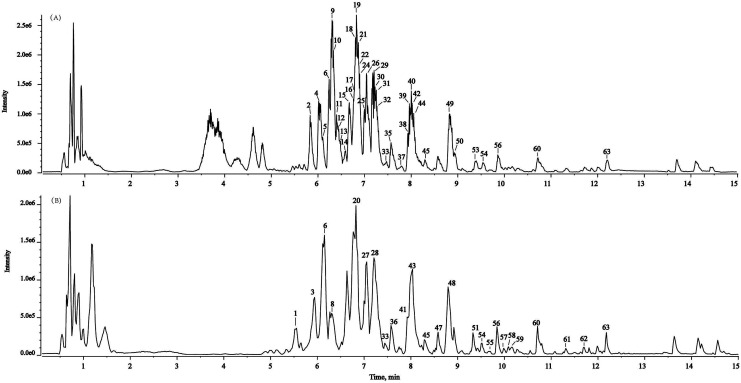
Representative base peak chromatogram (BPC) of Duoju samples acquired in IDA mode and SWATH mode. (A) IDA mode. (B) SWATH mode.

In the present study, a powerful method (SIBDV) based on identical LC and a Triple TOFTM 5600+ mass spectrometer was developed to bi-directionally verify large amounts of data. In particular, the independent data acquisition technology of SWATH was developed with variable Q1 isolation windows and different CEs.^40^ SWATH can therefore easily enhance the hit rate of the MS/MS spectrum and discover relevant ions in low-concentration ranges, complementing and expanding the data collected through IDA. For instance, the *m*/*z* 285.04155 peak of kaempferol in Taiju was identified by SWATH but was not detected in IDA mode due to the low ion response. As shown in Fig. 2 and 3, the base peak chromatograms (BPC) of Taiju and Duoju were obtained *via* UHPLC-QTOF-MS/MS using each of the two methods. It can be concluded that this method is not only efficient for identifying unknown trace compounds, but also improves the accuracy of the MS2 spectrum and subsequent exploration of chemical markers.

### 3.2 Identification of the constituents in Taiju and Duoju

#### 3.2.1 Analysis of crucial changed volatile metabolites in Taiju and Duoju

**Fig. 4:**
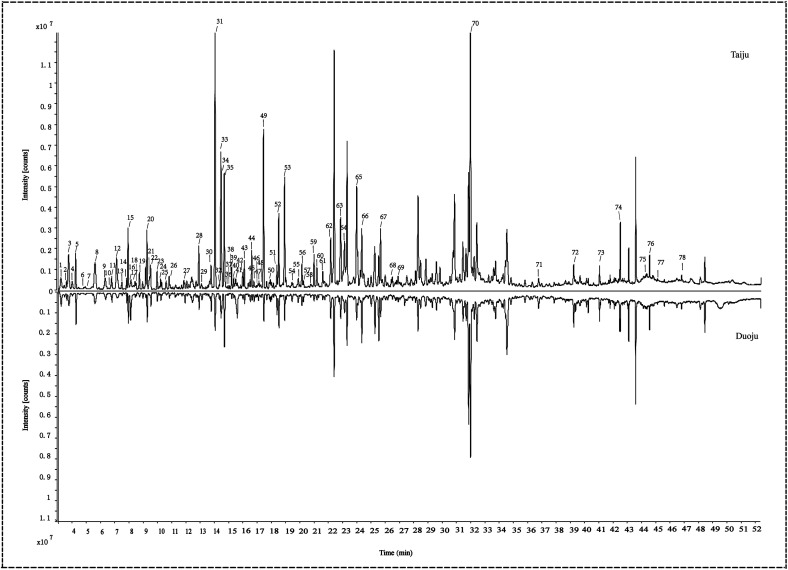
Total ion chromatograms of essential oils identified in Taiju and Duoju using GC-MS.

As characteristic active flavoring ingredients in Taiju and Duoju, volatile oils have become the main research object in volatile metabolites analysis. GC-MS was used to analyze the volatile oils in Taiju and Duoju samples collected at different development stages and to determine the metabolic profiles of these samples based on total ion chromatograms (TIC). The GC-MS chromatograms of Taiju and Duoju are shown in Fig. 4. In the present study, 78 volatile oils were tentatively identified based on the mass spectral library, retention indices, and the literature related to the extracts of Taiju and Duoju. The detailed information for the 78 volatile compounds, including retention times, CAS ID, molecular formula, and identification, are summarized in Table 1. Interestingly, 78 volatile oil compounds were present in both Taiju and Duoju, while the contents obviously were different. Therefore, the flavor characteristics of HJ are related to the contents of volatile oils. It is necessary to find specific markers in Taiju and Duoju by using the technique of multivariate statistical analysis.

**Table 1:** The total volatile components from the *Chrysanthemum morifolium*

No.	*t*_R_ (min)	CAS	Molecular formula	Identification
1	3.188	105-46-4	C_6_H_12_O_2_	*sec*-Butyl acetate
2	3.431	110-19-0	C_6_H_12_O_2_	Isobutyl acetate
3	3.825	637-78-5	C_6_H_12_O_2_	Isopropyl propionate
4	4.235	123-86-4	C_6_H_12_O_2_	Butyl acetate
5	4.458	624-24-8	C_6_H_12_O_2_	Valeric acid methyl ester
6	5.495	108-38-3	C_8_H_10_	*meta*-Xylene
7	5.554	95-47-6	C_8_H_10_	*ortho*-Xylene
8	5.585	3387-41-5	C_10_H_16_	Sabinene
9	6.295	13466-78-9	C_10_H_16_	Carene (delta-3-)
10	6.313	106-42-3	C_8_H_10_	*para*-Xylene
11	6.75	5989-27-5	C_10_H_16_	(+)-Limonene
12	7.114	13877-91-3	C_10_H_16_	(*Z*)-β-Ocimene
13	7.363	1122-58-3	C_7_H_10_N_2_	4-Dimethylaminopyridine
14	7.447	99-85-4	C_10_H_16_	Terpinene (gamma-)
15	7.983	108-94-1	C_6_H_10_O	Cyclohexanone
16	8.003	98-82-8	C_9_H_12_	Isopropylbenzene
17	8.393	586-62-9	C_10_H_16_	Terpinolene
18	8.464	111-66-0	C_8_H_16_	1-Octene
19	8.924	622-96-8	C_9_H_12_	*para*-Ethyltoluene
20	9.135	2437-95-8	C_10_H_16_	β-Pinene
21	9.419	470-67-7	C_10_H_18_O	1,4-Cineole
22	9.492	1998/6/6	C_10_H_14_	*tert*-Butylbenzene
23	9.941	2437-95-8	C_10_H_16_	α-Pinene
24	10.098	776-76-1	C_13_H_14_Si	Methyldiphenylsilane
25	10.105	3777-69-3	C_9_H_14_O	2-Pentylfuran
26	10.186	100-52-7	C_7_H_6_O	Benzaldehyde
27	11.884	112-31-2	C_10_H_20_O	Decanal
28	12.888	124-18-5	C_10_H_22_	Decane
29	13.05	14593-43-2	C_10_H_12_O	Allyl benzyl ether
30	13.712	29135-27-1	C_12_H_18_O_2_	Verbenyl acetate
31	13.996	38301-80-3	C_10_H_14_O	Chrysanthenone
32	14.18	629-50-5	C_13_H_28_	Tridecane
33	14.405	507-70-0	C_10_H_18_O	Borneol
34	14.421	89-49-6	C_12_H_20_O_2_	Isopulegyl acetate (equatorial)
35	14.609	19822-67-4	C_10_H_16_O	Karahanaenone
36	14.83	1960/12/8	C_8_H_10_O	2-Phenylethyl alcohol
37	15.003	16491-36-4	C_10_H_18_O_2_	*cis*-3-Hexenyl butyrate
38	15.16	20777-49-5	C_12_H_20_O_2_	Dihydrocarvenyl acetate (axial)
39	15.168	75684-65-0	C_12_H_20_O_2_	Dihydrocarvyl acetate
40	15.423	98-92-0	C_6_H_6_N_2_O	Nicotinamide
41	15.425	18486-69-6	C_10_H_14_O	Myrtenal
42	15.616	1120-21-4	C_11_H_24_	Undecane
43	16.094	20834-59-7	C_11_H_16_O	*p*-Cymen-8-ol
44	16.591	7111-29-7	C_12_H_18_O_2_	*cis*-l-Carvyl acetate
45	16.603	1134-95-8	C_12_H_18_O_2_	*trans*-l-Carvyl acetate
46	16.735	629-59-4	C_14_H_30_	Tetradecane
47	17.082	105-86-2	C_11_H_18_O_2_	Geranyl formate
48	17.093	138-87-4	C_10_H_18_O	β-Terpineol
49	17.41	50277-27-5	C_14_H_24_O_2_	Bornyl isobutyrate
50	17.805	99-93-4	C_8_H_8_O_2_	4′-Hydroxyacetophenone
51	18.095	4179-38-8	C_12_H_20_O	2-Octylfuran
52	18.337	112-40-3	C_12_H_26_	Dodecane
53	18.877	475-20-7	C_15_H_24_	Longifolene
54	19.449	629-62-9	C_15_H_32_	Pentadecane
55	19.759	112-17-4	C_12_H_24_O_2_	Decyl acetate
56	20.088	469-61-4	C_15_H_24_	α-Cedrene
57	20.12	73744-93-1	C_15_H_24_	Sesquiphellandrene (beta-)
58	20.135	6980-46-7	C_15_H_24_	Amorphene (gamma)
59	20.212	10486-19-8	C_13_H_26_O	Tridecanal
60	21.033	6753-98-6	C_15_H_24_	α-Caryophyllene
61	21.139	30021-74-0	C_15_H_24_	γ-Muurolene
62	22.116	10219-75-7	C_15_H_24_	Eremophilene
63	22.729	629-73-2	C_16_H_32_	Hexadecane
64	23.053	37839-63-7	C_15_H_24_	Germacrene-d
65	23.937	15423-57-1	C_15_H_24_	Germacrene-b
66	24.459	2221-76-3	C_14_H_20_O	Khusitone
67	25.622	24851-98-7	C_13_H_22_O_3_	Cepionate
68	26.107	629-78-7	C_17_H_36_	Heptadecane
69	26.46	639-99-6	C_15_H_26_O	Elemol
70	31.925	19892-19-4	C_16_H_20_O_4_	Deoxysericealactone
71	36.583	112-95-8	C_20_H_42_	Eicosane
72	39.319	629-92-5	C_19_H_40_	Nonadecane
73	41.061	629-97-0	C_22_H_46_	Docosane
74	42.415	629-94-7	C_21_H_44_	Heneicosane
75	44.084	630-01-3	C_26_H_54_	Hexacosane
76	44.52	638-67-5	C_23_H_48_	Tricosane
77	45.552	646-31-1	C_24_H_50_	Tetracosane
78	46.765	629-99-2	C_25_H_52_	Pentacosane

#### 3.2.2 Analytic strategy for rapidly distinguishing flavonoids in Taiju and Duoju using UPLCMS/MS

As one of the necessary active constituents in HJ herbs, flavonoids became the main research object in this study. Based on the fragmentation pathways of reference compounds, the flavonoid cleavage patterns were summarized in a comprehensive study. In this experiment, 19 flavonoid reference standards were selected and divided into three types, involving flavone/flavanone aglycones (apigenin, luteolin, naringenin, diosmetin, acacetin, kaempferol, quercetin, isorhamnetin), flavonoid-*C*-glycosides (vitexin, schaftoside) and flavonoid-*O*-glycosides (astragalin, quercitrin, isoquercitrin, kaempferide-7-*O*-glucoside, quercimeritrin, baicalin, rutin, apigenin-7-*O*-glucuronide and hesperidin). A summary of the fragmentation rules for ions served as the basis for identification. In the negative ion mode, the retro-Diels–Alder (RDA) cleavage reaction readily occurs at the 1.3 bonds of the C ring of the flavonoid/flavanone aglycones, producing the characteristic fragment ions of *m*/*z* 151 [1,3A]^−^, *m*/*z* 117 [1,3B]^−^ (apigenin, Fig. S1A†) and *m*/*z* 133 [1,3B]^−^ (luteolin, Fig. S1B†). RDA cleavage appeared to produce *m*/*z* 107 [0,4A]^−^ for flavonoids at the 0.4 bond (quercetin, Fig. S1C†). Furthermore, all flavonoid glycosides readily lose neutral molecules in the negative ion mode, such as H_2_O, CO_2_, C_2_H_2_O, C_3_O_2_, and C_6_H_6_O_2_, corresponding to fragment ions of 18, 44, 42, 68, and 110 Da, respectively. *O*-Glycosides are unstable, undergoing both cleavage and H-rearrangement at the glycosidic-*O*-linkages and leading to the elimination of saccharide residues. In the reference substances astragalin, kaempferol-7-*O*-β-d-glucopyranoside and rutin, we found that flavonoid-*O*-glycosides were substituted by monosaccharide and disaccharide, which completely remove the sugar molecule to obtain the aglycone ion [Y_0_]^−^ and the radical aglycone ion [Y_0_ − H]^−^. The aglycone ion [Y_0_]^−^ still undergoes RDA cleavage and the radical aglycone ion [Y_0_ − H]^−^ easily loses the neutral molecule. The detailed cleavage regularity is shown in Fig. S2.† An acid-resistant C–C bond links the sugar to the flavonoid nucleus in flavonoid *C*-glycosides. As illustrated by the cases of vitexin and schaftoside (Fig. S3†), the molecular ion peak [M − H]^−^ undergoes glucogenic ring cleavage at 0,4X1−, 0,3X1−, 0,2X1−, and loses C_2_H_4_O_2_ (−60 Da), C_3_H_6_O_3_ (−90 Da), and C_4_H_8_O_4_ (−120 Da) respectively. Based on the above patterns, the number of oligosaccharide chains was inferred and the structures were further accurately determined, which are the guiding principles for analyzing and identifying the structures of flavonoids using UHPLC-QTOF-MS/MS.

Eventually, 63 flavonoids and their isomers were identified from the extracts of Taiju and Duoju, including flavonoid, flavonol, dihydroflavonoid, dihydroflavonol, and isoflavone. The details are summarized in Table 2, including chemical formulas, identification, retention times, calculated and experimental *m*/*z* values, ppm error, and characteristic MS/MS fragment ions. Compounds 5, 6,11, 12, 18, 23, 27, 29, 30, 33, 37, 47, 48, 54, 55, 57, 58, 59, and 63 were unambiguously identified as schaftoside, rutin, isoquercitrin, kaempferide-7-*O*-glucoside, quercimeritrin, hesperidin, astragalin, apigenin 7-*O*-glucuronide, baicalin, quercitrin, vitexin, luteolin, quercetin, apigenin, naringenin, kaempferol, diosmetin, isorhamnetin and acacetin by comparison with the standards, respectively. The composition of the other 44 flavonoids was tentatively determined based on the fragmentation regularity and comparison with data from previous publications.

**Table 2:** Identification of flavonoids by UHPLC-Q-TOF/MS

No.	Formula	*t*_R_ (min)	Identification	*m*/*z* [M − H]^−^	Error (ppm)	MS/MS fragments	Source
1	C_21_H_20_O_11_	5.61	Cynaroside	447.1151	1.3	447.0907 327.0437 285.0391 151.0023	T,D
2	C_28_H_32_O_17_	5.63	Isorhamnetin-3,7-*O*-diglucoside	639.1573 2	1	639.1251 463.0907 300.0281 271.0249 151.00	D
3	C_21_H_20_O_13_	5.69	Myricetin-3-*O*-galactoside	479.08388	1.6	479.0842 303.0573 285.0405 117.0186 125.0243	T,D
4	C_27_H_30_O_15_	5.8	Vicenin II	593.15378	4.4	593.1517 503.1183 473.1080 353.0664 325.0712	T,D
5^a^	C_26_H_28_O_14_	5.96	Schaftoside	563.14182	2.1	563.1422 473.1083 443.0973 353.0655 175.0601	T,D
6^a^	C_27_H_30_O_16_	6.23	Rutin	609.14668	0.9	609.1484 447.0943 301.0353 300.0267 257.0451 151.0043	T,D
7	C_21_H_20_O_12_	6.42	Myricitrin	463.08924	2.2	463.0892 343.0467 300.0285 301.0364 151.0042	T
8	C_21_H_20_O_12_	6.43	Quercetin-3-*O*-β-d-galactopyranoside	463.09028	4.5	463.0886 301.0360 287.0517 257.2453 151.0029	T,D
9	C_21_H_22_O_11_	6.44	Eriodictyol-7-glucoside	449.10924	0.7	447.0910 327.0491 285.0398 284.0318 151.0025	T,D
10	C_21_H_20_O_11_	6.45	Isoorientin	447.09486	3.5	447.0907 327.0484 285.0391 151.0023	T,D
11^a^	C_21_H_20_O_12_	6.46	Isoquercitrin	463.09028	4.5	463.0920 300.0278 271.0248 151.0034	D
12^a^	C_21_H_20_O_11_	6.48	Kaempferide-7-*O*-glucoside	447.09486	3.5	447.0923 285.0379 284.0318 151.0027	T,D
13	C_21_H_20_O_11_	6.5	Luteolin-7-*O*-β-d-galactoside	447.09486	3.5	447.0923 327.0490 285.0397 151.0027	T,D
14	C_21_H_18_O_13_	6.57	Quercetin-3-*O*-glucuronide	477.0678	0.7	477.0695 301.0357 151.0032	T,D
15	C_27_H_30_O_14_	6.61	Rhoifolin	577.15702	1.3	577.1553 269.0456 151.0064	T,D
16	C_27_H_30_O_14_	6.62	Kaempfero-3-*O*-xyloside	577.15762	2.3	577.1585 269.0464 151.0008 159.0437	T,D
17	C_21_H_18_O_12_	6.63	Luteolin-7-*O*-glucuronide	461.07414	3.5	461.0705 285.0399 151.0026	T,D
18^a^	C_21_H_20_O_12_	6.71	Quercimeritrin	463.09028	4.5	463.0866 287.0543 151.0029	D
19	C_21_H_20_O_13_	6.72	Gossypin	479.08388	1.6	479.0847 369.0460 303.0499 285.0401 271.0634 193.0141 133.0286	T,D
20	C_15_H_12_O_6_	6.74	Eriodictyol	287.05567	-1.5	287.0506 241.1529 151.0040 135.0456	T,D
21	C_24_H_22_O_15_	6.79	Quercetin-3-*O*-(6′′-*O*-malonyl)-glucoside	549.08976	2.1	549.1999 300.0274 301.0354 161,0240 151.0035	T,D
22	C_28_H_32_O_15_	6.8	Diosmin	607.16895	3.5	607.1683 299.0564 284.0324 151.0030	T,D
23^a^	C_28_H_34_O_15_	6.94	Hesperidin	609.18426	2.9	609.1861 301.0731 242.0576 164.0121	T,D
24	C_22_H_22_O_12_	6.97	Isorhamnetin-7-*O*-β-d-glucoside	477.10458	1.5	447.1058 431.0994 314.0437 315.0475 151.0041	T
25	C_21_H_20_O_10_	6.99	Apigenin-7-*O*-β-d-glucoside	431.0992	1.9	431.0976 311.0548 268.0373 269.0442 151.0023	T,D
26	C_24_H_22_O_14_	7	Luteolin-7-*O*-6"-malonylgalactoside	533.09557	3.5	489.1022 285.0399 175.0383 151.0026	T,D
27^a^	C_21_H_20_O_11_	7.03	Astragalin	447.09486	3.5	447.0942 285.0416 284.0334 256.0371 151.0032	T,D
28	C_15_H_12_O_6_	7.05	Carthamidin-4',5,7,8-tetrahydroxyflavanone	287.05567	-1.5	287.0506 241.1529 151.0040 135.0456	T,D
29^a^	C_21_H_18_O_11_	7.2	Apigenin-7-*O*-glucuronide	445.07891	2.9	445.0780 269.0465 175.0244 151.0031	T,D
30^a^	C_21_H_18_O_11_	7.3	Baicalin	445.07891	2.9	445.1714 269.0463 243.1236 113.0242	D
31	C_23_H_22_O_13_	7.32	Chrysin-7-*O*-β-d-glucuronide	505.10034	3.1	505.1019 445.0793 329.0678 175.0240 151.0033	T,D
32	C_22_H_20_O_13_	7.35	Isorhamnetin-3-glucuronide	491.08481	3.5	491.1118 315.0516 300.0274 287.0557 269.0454 151.0035	D
33^a^	C_21_H_20_O_11_	7.36	Quercitrin	447.09486	3.5	447.0943 271.0623 175.0249 151.0040	T,D
34	C_22_H_24_O_11_	7.42	Hesperetin-7-*O*-β-d-glucoside	463.12496	0.8	463.0917 301.0734 286.0484 151.0037	T,D
35	C_23_H_22_O_11_	7.53	6''-*O*-acetylgenistin	473.1113	5	473.1090 311.0547 268.0373 151.0021	T,D
36	C_15_H_10_O_5_	7.57	Genistein	269.04708	5.7	269.0448 225.0545 151.0025 117.0341	T,D
37^a^	C_21_H_20_O_10_	7.59	Vitexin	431.0992	1.9	431.0985 311.0544 268.0378 151.0030	T,D
38	C_16_H_12_O_7_	7.68	3-*O*-methylquercetin	315.05153	1.6	315.1270 300.0286 269.0463 225.0553 151.0037	T,D
39	C_22_H_22_O_12_	7.69	Isorhamnetin-3-*O*-galactoside	477.10521	2.9	477.1070 301.0728 175.0243 113.0248	T,D
40	C_25_H_26_O_14_	7.79	Limocitrin-7-*O*-(6''-acetyl-β-d-glucopyranoside)	549.12665	3	503.1189 299.0559 284.0319 151.0021	T,D
41	C_16_H_12_O_6_	7.80	Hydroxygenkwanin	299.05687	2.5	284.0331 256.0372 151.0031	T,D
42	C_16_H_14_O_6_	7.96	Hesperetin	301.07236	2	301.0729 286.0467 242.0568 151.0037	T,D
43	C_16_H_12_O_5_	7.97	5,4'-dihydroxy-7-methoxy isoflavones	283.06284	5.8	283.0611 268.0385 151.0034	T,D
44	C_36_H_28_O_15_	8.29	Ochnaflavone-7-*O*-β-d-glucopyranoside	699.13652	1.4	699.1480 537.1045 375.0731 179.0350	T,D
45	C_23_H_22_O_11_	8.32	Apigenin-7-*O*-glucuronide-6'-ethyl ester	473.1113	5	473.1080 268.0368 269.0435 151.0022	T,D
46	C_23_H_24_O_12_	8.58	Tricin-7-β-d-glucopyranoside	491.11984	0.7	283.0625 268.0379 151.0034	T
47^a^	C_15_H_10_O_6_	8.59	Luteolin	285.04217	6	285.0402 241.0489	T,D
48^a^	C_15_H_10_O_7_	8.67	Quercetin	301.03562	0.8	301.0356 273.0395 178.9986 151.0040 121.0293	T,D
49	C_22_H_20_O_11_	8.89	Wogonin-5-*O*-glucoside	459.09542	4.6	459.0937 283.0618 268.0379 175.0239 113.0243	T,D
50	C_22_H_20_O_11_	9.01	Apigenin-7-*O*-methylglucuronide	459.09542	4.6	459.0963 283.0626 268.0390 175.0255 113.0246	T,D
51	C_16_H_12_O_5_	9.24	Calycosin	283.06284	5.8	268.0378 240.0416 151.0029	T,D
52	C_22_H_28_O_11_	9.29	Prim-*O*-glucosylcimifugin	467.1546	-2.8	467.1883 313.1632 159.0304 161.0454	T,D
53	C_15_H_12_O_4_	9.44	Liquiritigenin	255.06649	0.8	255.0654 213.0536 151.0031 211.0741	D
54^a^	C_15_H_10_O_5_	9.61	Apigenin	269.04708	5.7	269.0456 225.0545 151.0028 117.0343	T,D
55^a^	C_15_H_12_O_5_	9.67	Naringenin	271.06102	-0.6	271.0585 227.0674 151.0033 119.0504	T,D
56	C_17_H_14_O_7_	9.75	5-Hydroxy-7,4′-dimethoxyflavonoid quercetin	329.06765	3	329.0659 314.0426 299.0194 271.0244 227.0343 151.0029	T,D
57^a^	C_15_H_10_O_6_	9.84	Kaempferol	285.04155	3.8	285.0402 239.0364 257.0459 229.0495 151.0029 133.0293	T,D
58^a^	C_16_H_12_O_6_	9.86	Diosmetin	299.05687	2.5	284.0372 256.0375 227.0342 151.0030	T,D
59^a^	C_16_H_12_O_7_	10.03	Isorhamnetin	315.05162	1.9	315.0516 300.0281 271.0236 151.0029	T,D
60	C_15_H_12_O_4_	10.83	Isoliquiritigenin	255.06649	0.8	255.2329 211.0757 143.0494	D
61	C_15_H_10_O_5_	11.24	Baicalein	269.04708	5.7	269.0456 225.0535 151.0035 117.0340	T,D
62	C_18_H_16_O_7_	11.51	Eupatilin	343.08346	3.3	343.0805 328.0583 313.0350 270.0164	T,D
63^a^	C_16_H_12_O_5_	12.22	Acacetin	283.06284	5.8	283.0612 268.0385 239.0349 151.0030	T,D

a Reference standards.

Among the 63 flavonoids identified, there are 53 common components in Taiju and Duoju. However, myricitrin, isorhamnetin-7-*O*-β-d-glucoside and ricin-7-β-d-glucopyranoside were only found in Taiju. It has anti-inflammatory, analgesic, and choleretic pharmacological effects. Seven components were only found in Duoju, including isorhamnetin-3,7-*O*-diglucoside, isoquercitrin, quercimeritrin, baicalin, isorhamnetin-3-glucuronide, liquiritigenin and isoliquiritigenin. It can be used as a food additive and anti-inflammatory drug. The results indicate that there are differences in the pharmacological effects of Taiju and Duoju. Therefore, a statistical analysis was necessary to find the compounds that contribute most to the differences between the sensory characteristics and health effects.

### 3.3 Chemometric statistical analysis

#### 3.3.1 Search for volatile markers using PCA and OPLS-DA analysis

**Fig. 5:**
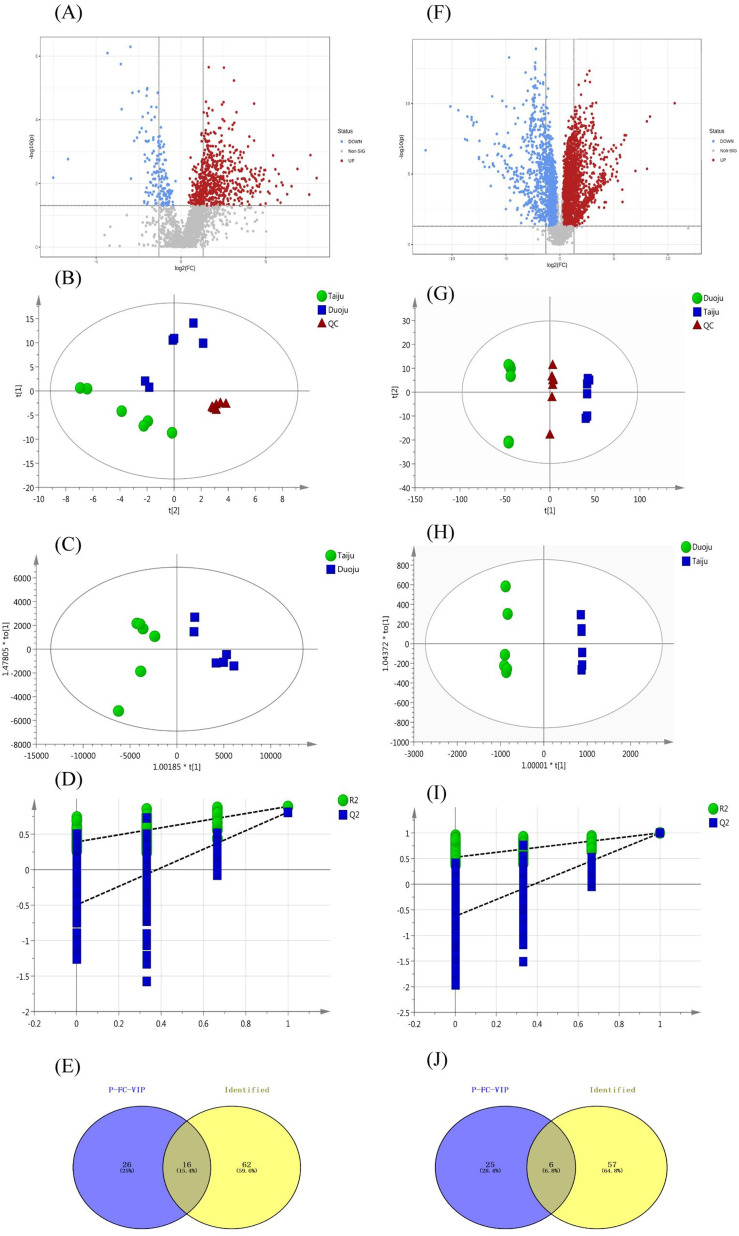
Multivariate statistical analysis of HJ in different harvest periods. (A) Volcano for GC-MS. (B) PCA score plot in GC-MS. (C) OPLS-DA score plot in GC-MS. (D) 200 permutations plot in GC-MS. (E) Venn diagram for screening marker metabolites in GC-MS. (F) Volcano in LC-MS. (G) PCA score plot in LC-MS. (H) OPLS-DA score plot in LC-MS. (I) 200 permutations plot in LC-MS, (J) Venn diagram for screening marker metabolites in LC-MS.

To obtain a clear overview of the compositional characteristics of the HJ herbs at different harvest times, a matrix model of unsupervised PCA was constructed to estimate the degree of separation. The metabolites with an FC value > 1.2 or <0.8 and *p* < 0.05 in the volcano plot from the MetaboAnalyst5.0 platform were screened primarily for chemometric statistical analysis (Fig. 5A). The PCA score plot in the negative ion mode is shown in Fig. 5B. The first and second principal components after dimensionality reduction using the PCA algorithm are represented by the *x*- and *y*-axes, respectively. Different groups are represented by dots of different colors and shapes. There is an obvious state of aggregation within the QC sample group, reflecting the accuracy of the data analysis and reliability of the results. In addition, the PCA score plot shows that samples were grouped into three distinct groups, which were considered to indicate statistically significant differences.

A PCA model with R2X = 0.758 and Q2 = 0.692 was proven to be reliable, and further stoichiometric statistics based on supervised OPLS-DA were used to create a better differentiation between the two groups separately. The OPLS-DA model was developed, and the R2Y and Q2 of the model were 0.907, and 0.999, respectively. As shown in Fig. 5C, the results satisfy R2Y > 0.5 and Q2 > 0.3. In addition, Fig. 5D shows the 200 times permutation model, which shows that Q2 is less than 0. This indicates that the model was not over-fitted and was reliable and accurate. Afterward, the compounds with VIP > 1.0 were screened as potential markers. Finally, 46 metabolites satisfying the three conditions (FC of >1.2 or <0.8, *p* < 0.05, and VIP > 1) were screened. As shown in Fig. 5E, 16 volatile metabolites were considered to be marker metabolites based on comparison with the identified metabolites in Taiju and Duoju. The detailed values are recorded in Table 3. The 16 metabolites corresponded to components 12, 20, 31, 33, 40, 41, 44, 49, 53, 62, 65, 67, 70, 74, 76 and 78 of the identified volatile oils.

**Table 3:** 16 specific markers of the essential oils

Taiju–Duoju						
No.	Identification	FC	*P*	VIP	Trend	AUC
1	(*Z*)-β-Ocimene	0.12931	5.45 × 10^−7^	2.22705	↑	1.000
2	β-Pinene	0.2037	0.00019869	1.1042	↑	1.000
3	Chrysanthenone	0.18087	1.27 × 10^−5^	3.40872	↑	—
4	Borneol	0.25698	8.79 × 10^−6^	3.73499	↑	1.000
5	Nicotinamide	0.48913	0.00067825	1.41383	↑	—
6	Myrtenal	0.51015	0.0076246	1.14561	↑	—
7	*cis*-l-Carvyl acetate	0.25342	9.81 × 10^−6^	1.17883	↑	1.000
8	Bornyl isobutyrate	0.24891	1.57 × 10^−5^	2.64804	↑	1.000
9	Longifolene	0.40082	0.00049672	1.41966	↑	—
10	Eremophilene	0.3029	9.39 × 10^−5^	1.28276	↑	—
11	Germacrene-b	0.40598	0.00044259	1.4376	↑	—
12	Cepionate	0.61752	0.011381	2.36112	↑	—
13	Deoxysericealactone	0.36528	0.012746	1.36153	↑	—
14	Heneicosane	20.352	0.00044876	1.19437	↓	—
15	Tricosane	12.117	0.0040324	1.33354	↓	—
16	Pentacosane	27.87	0.007891	1.27865	↓	—

#### 3.3.2 Search for flavonoid compound markers

After serial data pretreatment using Progenesis QI software, an alignment multivariate dataset was obtained, which was screened with the criteria of an FC of >1.2 or <0.8 and *p* < 0.05 using MetaboAnalyst5.0 platform, preliminary (Fig. 5F). In UHPLC-QTOF-MS, the obvious separation of PCA shows that there was a significant difference between Taiju and Duoju (Fig. 5G). The results of the OPLS-DA and 200 permutations plot suggested that the model was not overfitted (Fig. 5H–I). It was reliable and accurate (R2Y = 0.999 and Q2 = 0.994, respectively). The final screening resulted in 31 metabolites that satisfied the three criteria (FC of >1.2 or <0.8, *p* > 0.05, VIP > 3.0). As shown in Fig. 5J, we considered six of these to be marker metabolites based on comparison with the identified flavonoid metabolites in Taiju and Duoju. The detailed values are shown in Table 4. The six metabolites were hydroxygenkwanin, luteolin, apigenin, diosmetin, isorhamnetin and eupatilin, which correspond to components 41, 47, 54, 58, 59 and 62 of the identified flavonoids, respectively.

**Table 4:** Specific markers of flavonoids from HJ and their binding energies against liver cancer

No.	Identification	FC	*P*	VIP	AUC	Binding energy (kcal mol^−1^) of CDK1	Binding energy (kcal mol^−1^) of PLK1
1	Luteolin	0.34674	3.63 × 10^−10^	10.2725	1.000	−6.27	−7.11
2	Apigenin	0.65486	2.27 × 10^−5^	4.0180	1.000	−6.77	−5.37
3	Diosmetin	0.48228	8.07 × 10^−9^	3.7749	1.000	−6.46	−7.44
4	Isorhamnetin	0.038783	5.51 × 10^−14^	3.3627	1.000	−6.44	−6.07
5	Eupatilin	1.3659	3.28 × 10^−5^	3.3002	1.000	−6.01	−6.34
6	Hydroxygenkwanin	0.18634	7.91 × 10^−11^	3.1115	1.000	−6.48	−6.00

#### 3.3.3 Visualization of marker metabolites

A total of 22 differential markers were obtained in the OPLS-DA model by requiring VIP > 1 (or VIP > 3) and *P* < 0.05. Of these, 16 components were volatile ingredients, and 6 components had a flavonoid composition. However, there is still a lack of clarity as to the distribution of the identified differential markers. By using a heat map, the content of different markers in the two herbs can be visualized using color, which helps to better understand the distribution changes. The data set consisted of the approximate peak areas of 22 components, and a heat map with hierarchical clustering was generated from this data set in MetaboAnalyst 5.0 software. The hierarchical cluster analysis is statistically significant. The rows represent the type of compound, and the columns represent the batches. The names of the marker metabolites and the sample groups are shown on the right and the top of the graph, respectively. Additionally, the color represents the relative content of metabolites; a more dark-red color indicates a higher content; and a more blue color indicates lower content.

**Fig. 6:**
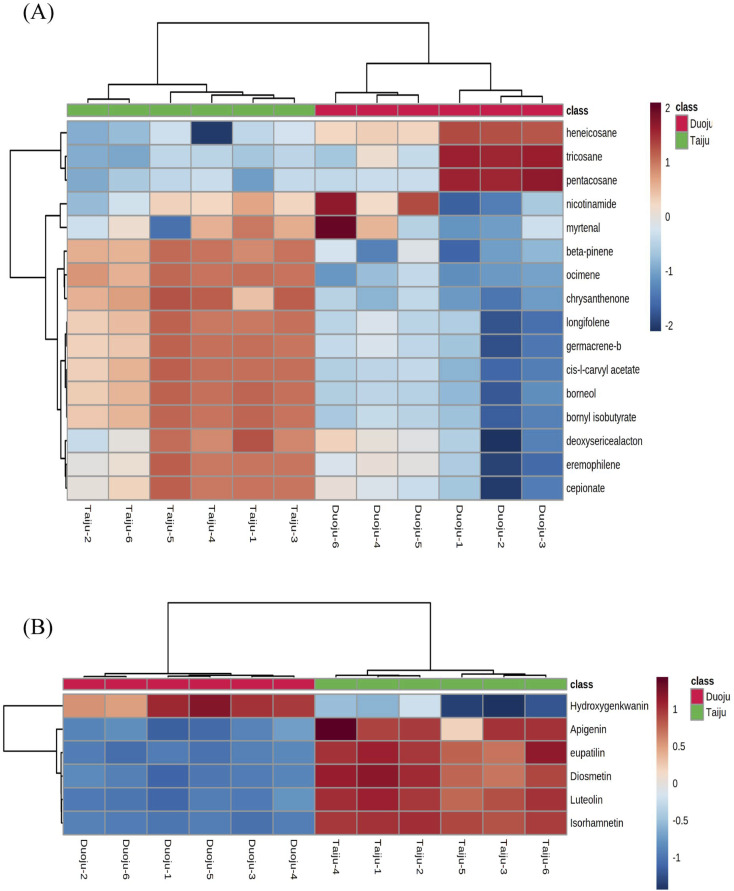
The hierarchical cluster analysis of marker metabolites. (A) Heat map based on the relative abundance of 16 volatile markers in GC-MS. (B) Heat map of 6 flavonoid markers in UPLC-Q/TOF-MS.

As shown in Fig. 6A, clear and visual differences and associations between the 16 volatile oil marker metabolites were shown by the heat map. Among the 16 markers, thirteen ingredients (ocimene, beta-pinene, chrysanthenone, borneol, nicotinamide, myrtenal, l-carvyl acetate, bornyl isobutyrate, longifolene, eremophilene, germacrene-b, cepionate, deoxysericealactone) showed high relative contents in Taiju. The other three ingredients including heneicosane, tricosane and pentacosane, have higher contents in Duoju. Interestingly, we found that the three ingredients in Duoju are odorless. Furthermore, we also found that the 13 fragrance components with higher content in Taiju were mainly composed of alcohols, terpenes and esters. The 13 volatile markers were categorized into 5 types: grassy scent (ocimene), woody scent (longifolene), jasmine of the valley scent (cepionate), spearmint scent (l-carvyl acetate) and aromatic flavor (the compounds' aroma characteristics were taken from http://www.thegoodscentscompany.com/search2.html).^41^ Moreover, the above ingredients are fragrant spices, which are commonly used in spice mixtures, cosmetics and food.^42^ The content levels of the above ingredients were significantly higher in Taiju than in Duoju. A significant difference between the specific floral scent characteristics in Taiju and Duoju might be its sweet and floral aroma. It helps to verify the result that the aroma of Taiju is more rich and more mellow in sensory evaluation. Therefore, the harvesting period affects the aroma of HJ; Taiju has a stronger and mellow aroma. The results could also explain the popularity and higher price of Taiju.

In addition, six flavonoids (luteolin, apigenin, diosmetin, isorhamnetin, eupatilin and hydroxygenkwanin) were screened as specific markers to distinguish Taiju and Duoju. Their affiliation and content are shown in Fig. 6B. We found that these components existed in both Taiju and Duoju, indicating that HJ plays a functional food role throughout the growth period. However, the two have significantly different contents. Only one compound, hydroxygenkwanin, showed higher expression in Duoju, while the other five compounds were highly expressed in Taiju, with the most prominence for luteolin. However, the significantly different contents indicated that the flavonoids of HJ reached their peak during the Taiju growth stage and were then metabolized as the growth period progressed.

#### 3.3.4 The establishment and evaluation of the diagnostic model

**Fig. 7:**
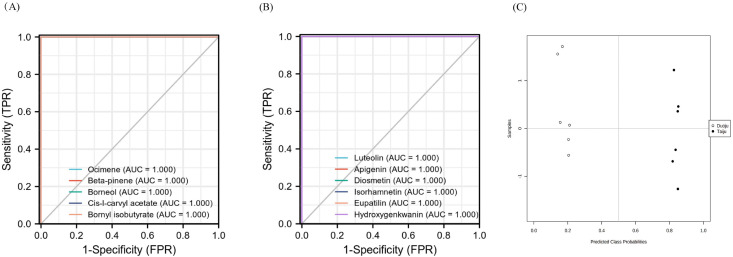
Receiver operating characteristic curve (ROC) plot and evaluation of the predictive power of the diagnostic panel. (A) ROC in CG-MS. (B) ROC in UHPLC-Q-TOF-MS. (C): Diagnostic performance of classifier modeled by RF algorithm.

Based on the receiver operator characteristic (ROC) curve, 22 chemical markers (16 volatile oils and 6 flavonoids) were evaluated. The AUC (area under the ROC curve) of 11 chemical markers was 1, indicating their credibility as potential diagnostic chemical markers. As shown in Fig. 7A and B, the ROC plots of the 11 diagnostic chemical markers were obtained. It indicated that the 11 diagnostic markers had good performance in recognizing Taiju and Duoju. These 11 markers were ocimene, beta-pinene, borneol, l-carvyl acetate, bornyl isobutyrate, luteolin, apigenin, diosmetin, isorhamnetin, eupatilin and hydroxygenkwanin.

To assess the predictive power of the diagnostic panel, the analyzed samples (*n* = 28) were selected to compose a validation set. Among them, 16 samples of unknown classification, including eight Taiju and eight Duoju samples, were labeled and disordered for analysis. After performing the Tester module and RF algorithm analysis using MetaboAnalyst5.0, there was a separation trend between the Taiju and Duoju groups (Fig. 7C). In addition, the 16 unknown samples were classified as Taiju and Duoju, and the groupings were consistent with the label (Table S2†). Therefore, the results indicate that this diagnostic model has good predictive ability and the 11 components have a good ability to distinguish between Taiju and Duoju.

### 3.4 Bioinformatics analysis

Various studies have shown that HJ has curative effects on liver diseases, and flavonoids are the active components of this effect.^43^ Therefore, the flavonoid differential metabolites with liver cancer targets were screened for molecular docking validation to verify whether the different harvesting stages of HJ have different anti-cancer activities.

**Fig. 8:**
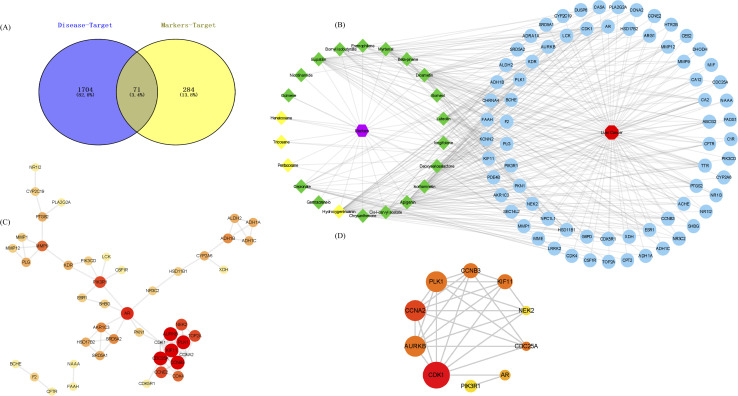
Bioinformatics analysis prediction for HJ treatment of liver diseases. (A) Venn diagram of HJ and hepatoma intersection targets. (B) Compound-target-disease network. (C) PPI network of potential targets. (D) Map of the hub genes screened from PPI using CytoHubba.

**Fig. 9:**
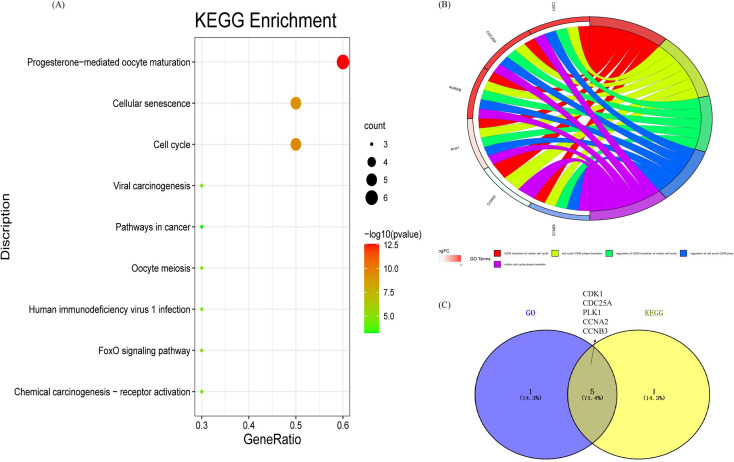
Distribution of functional enrichment analysis. (A) Bubble chart of KEGG pathways. (B) Detailed relationship between differentially expressed genes and major pathways annotated by GO shown using a Circos graph. (C) Intersection of the KEGG and GO biological processes.

The GSE84402 dataset was downloaded from the GEO database, |log FC| > 1 and *p* < 0.05 were set as the screening criteria, and the genes expressed in healthy human liver tissue (14) and liver cancer tissue (14) were selected as research objects. There were 690 up-regulated and 1085 down-regulated differential genes (DEGs) (see Appendix IV for details†), and a volcano map of differential genes was created. In Fig. S4,† log FC > 0 (red) indicates an up-regulated gene and log FC < 0 (green) represents a down-regulated gene in the experimental group. In addition, the 355 gene targets of differential flavonoid compositions were predicted by Pharmmapper and SwissTargetPrediction. A total of 1775 differential genes (DEGs) were obtained from the high-throughput microarray dataset, intersected with 355 targets of the drug to obtain 71 associated gene targets, and plotted as a Venn diagram (Fig. 8A). Then, the network and type files based on the corresponding properties were created and the component-target-disease network pharmacology diagram was constructed in Cytoscape (Fig. 8B). Diamonds represent markers, red hexagons represent diseases and blue circles represent associated genes. Next, PPI networks were formed by importing 71 intersecting genes into the String online website and containing 194 nodes; the results are shown in Fig. 8C. Topological analysis was then performed using the Cytohubba plug-in to obtain the key genes with TOP10 degree values, including CDK1, AURKB, CCNA2, PLK1, CCNB3, KIF11, NEK2, CDC25A, AR, and PIK3R1 (Fig. 8D). Nine pathways were obtained by KEGG enrichment analysis and plotted in bubble plots as shown in Fig. 9A. The *p*-values are indicated by color, and the number of genes is indicated by bubble size. The detailed information listed in Table S3† shows that the hsa04914 target gene pathway is the most associated, corresponding to 6 differential genes. Fig. 9B shows the chord plot of GO enrichment analysis, which was obtained using ggplot2 in RStudio with the five significantly enriched GO entries on the right and the corresponding six differential genes on the left (including four up-regulated genes and two down-regulated genes). As shown in Fig. 9C, five DEGs were obtained after taking the intersection of the differential genes of KEGG and GO, which are expected to be the potential therapeutic targets for patients with liver cancer.

### 3.5 Molecular docking

**Fig. 10:**
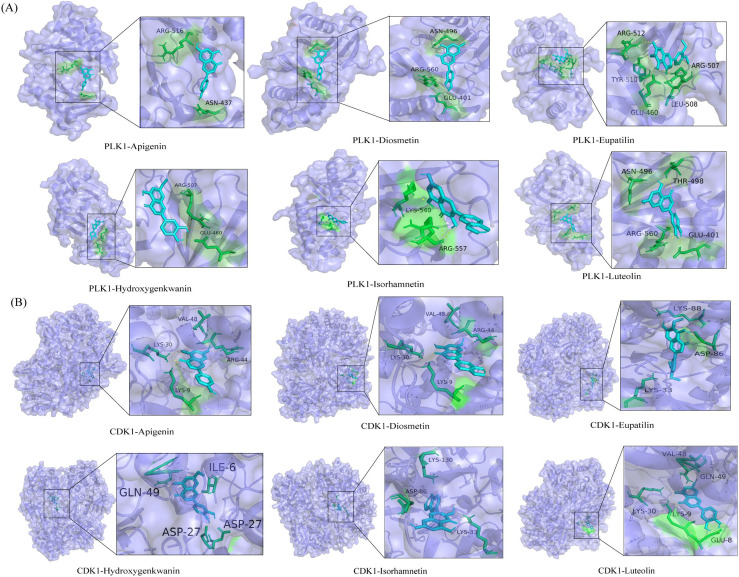
Molecular docking results of two anti-hepatoma core targets with flavonoid markers. (A) Lowest energy state of six flavonoid markers docked with PLK1. (B) Lowest energy state of six flavonoid markers docked with CDK1.

Molecular docking can simultaneously calculate binding energies and infer catalytic sites between receptors and ligands. PLK1 and CDK1 were selected as receptors because they were the core protein targets screened out using bioinformatics techniques. In addition, six flavonoid markers were used as ligands. Generally, the stabilities of conformations are positively correlated with their binding energies. The larger the absolute value of the binding energy, the more stable the conformation. The binding energies of the six flavonoid markers are listed in Table 4. A binding energy of <−5 kcal mol^−1^ indicates strong interaction ability.^44^ The results showed that all six biomarkers had binding energies ranging from −5.37 to −7.44 kcal mol^−1^, suggesting that the above-mentioned biomarkers have good affinity with core gene proteins. In general, these compounds were predominantly stable in the catalytic site due to electrostatic interactions, van der Waals forces, and hydrogen bonds.^45^ Some of the interaction forces between ligands and receptors are shown in Fig. 10. Among these compounds, diosmetin, with the lowest binding energy of −7.44 kcal mol^−1^, was completely enveloped by the active sites of PLK1. Some amino acid residues, including ASN-496, ARG-560 and GLU-401, contributed to the recognition *via* a combination of electrostatic interactions, van der Waals forces, and hydrogen bond interactions. Furthermore, apigenin and CDK1 formed hydrogen bonds at VAL-48, LYS-30, ARG-44 and LYS-9, causing them to form a stable complex (−6.77 kcal mol^−1^). This indicated that the six flavonoid markers will have higher biological activity.

In the present study, the molecular docking analysis was introduced to investigate the binding capacity of special markers with the core protein target of liver cancer. It suggested that the six flavonoid markers, including luteolin, apigenin, diosmetin, eupatilin, isorhamnetin and hydroxygenkwanin, had obvious advantages of protection against and treatment of liver diseases. Luteolin, apigenin and diosmetin are all natural flavonoids that have been used as dietary supplements. Luteolin alleviated hepatitis by regulating serum metabolites and intestinal microorganisms.^46^ Apigenin possessed anticancer, antioxidant, and anti-inflammatory effects, including inhibiting cell growth and proliferation in Hep3B cells.^47^ It was reported that diosmetin could inhibit the progression of cancer. Diosmetin also exhibited antitumor effects on HCC cells by inhibiting cell proliferation *via* cell cycle arrest and interfering with lipid metabolism.^48^ Additionally, eupatin inhibits hepatocellular carcinoma metastasis by intervening in MMP-2 mediated migration.^49^ Isorhamnetin may protect the liver by regulating NF-kB, Nrf2, and NLRP3 and reducing oxidative stress, inflammation, and scorch death.^50,51^ Additionally, hydroxygenkwanin has also been demonstrated to be active against liver cancer and validates its potential use as a therapeutic agent.^52^

Notably, only the content of hydroxygenkwanin was found to be significantly higher in Duoju. The other five flavonoids, including luteolin, apigenin, diosmetin, eupatilin and isorhamnetin, had higher contents in Taiju. Therefore, the inhibition of liver cancer activity of Taiju was higher than that of Duoju. The difference in pharmacological effects derived from different concentrations in markers may lead to a different emphasis in clinical medication. The anti-inflammatory and anti-hepatoma effects of flavonoid-rich in Taiju were more significant. Based on the above, in a TCM formula for heat-clearing and diet of HJ, Taiju may be a more effective and healthy material. It is suggested that Taiju may have a better health care effect and be a more effective health care medicine in the daily consumption of *Chrysanthemum* tea and TCM formulas for clearing heat and detoxifying, respectively. In the future, the pharmacological activity of markers needs to be validated to rationalize the clinical differences between Taiju and Duoju.

## 4 Conclusion

Through this research and analysis, we found that the relative content of volatile compounds with aromatic odor in Taiju increased significantly when the harvesting period was shortened, which may be the reason for the difference in flavor. The results of bioinformatics analysis combined with molecular docking showed that the flavonoid differential markers have good anti-liver cancer activity and are the potentially active ingredients. The flavonoid content in Taiju is significantly higher than that of Duoju, which causes a difference in medicinal effects. In conclusion, harvest time plays a remarkably important role in forming the metabolic profiles of HJ. The study should further facilitate the development of Taiju and Duoju food industries and its rational use in clinical applications, providing a powerful tool for the study of quality control.

## Author contributions

Mengxin Yang: methodology; data curation; software; writing – original draft; writing – review & editing. Xi Tian: data curation; methodology. Miaoting Zhang: software, visualization. Jinhuan Wei: software; supervision. Yukun Niu: writing – review & editing. Jiali Hou: investigation. Yiran Jin: resources; supervision; project administration. Yingfeng Du: funding acquisition; supervision; writing – review and editing.

## Conflicts of interest

The authors declare no conflict of interest.

## Supplementary Material

RA-012-D2RA05698D-s001
