# Supplementary material for: A holistic comparison of flavor signature and chemical profile in different harvesting periods of Chrysanthemum morifolium Ramat. based on metabolomics combined with bioinformatics and molecular docking strategy
Source: RSC Adv. 2022 Dec 7;12(54):34971–89. doi: 10.1039/d2ra05698d (PMC9728093; doi:10.1039/d2ra05698d)
Supplement: RA-012-D2RA05698D-s001 [file RA-012-D2RA05698D-s001.pdf]

Electronic Supplementary Material (ESI) for RSC Advances.
This journal is © The Royal Society of Chemistry 2022

Supplementary Materials

Title: A holistic comparison on flavor signature and chemical profile in different harvesting periods of *Chrysanthemum morifolium Ramat.* based on metabolomics combined with bioinformatics analysis and molecular docking strategy

Mengxin Yang[a], Xi Tian[a], MiaotingZhang[a], jinhuanWei[a], yukunNiu[a], JialiHou[a], YiranJin[b]**, YingfengDu[a]*

[a] Department of Pharmaceutical Analysis, School of Pharmacy, Hebei Medical University, Shijiazhuang, Hebei, 050017, P.R. China

[b] The Second Hospital of Hebei Medical University, Shijiazhuang, Hebei, 050000, P. R. China

*Corresponding author

*Yingfeng Du

Tel: +86-311-86265625; Fax: +86-311-86266419

E-mail address: yingfengdu@hotmail.com

**YiranJin

Tel: +86-311-86265625; Fax: +86-311-86266419

E-mail address: jinyiran@sohu.com

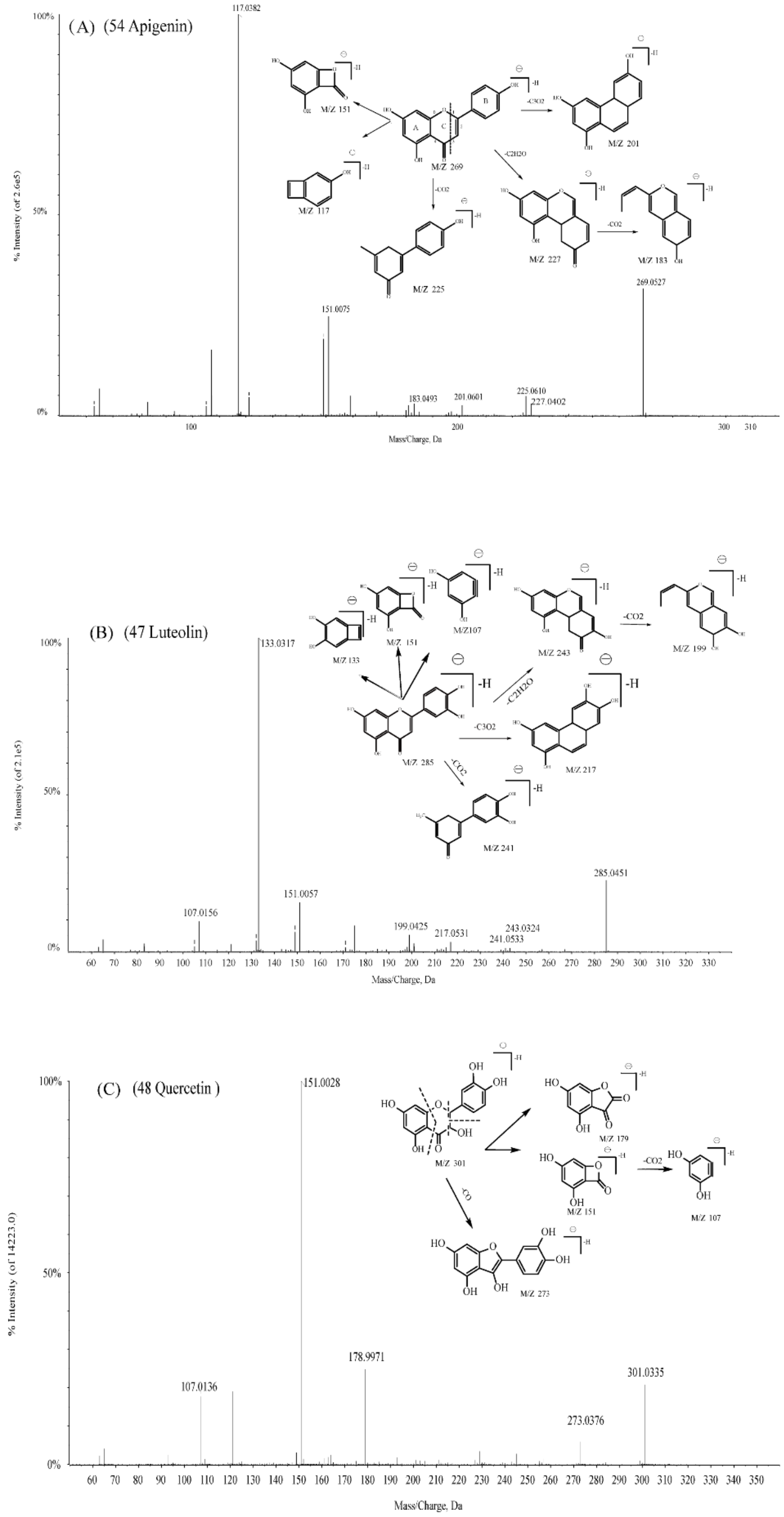

Fig.S1 The typical MS/MS spectrum of flavone/flavanone aglycones and their fragmentation pathways: (A) Apigenin, (B) Luteolin, (C) Quercetin.

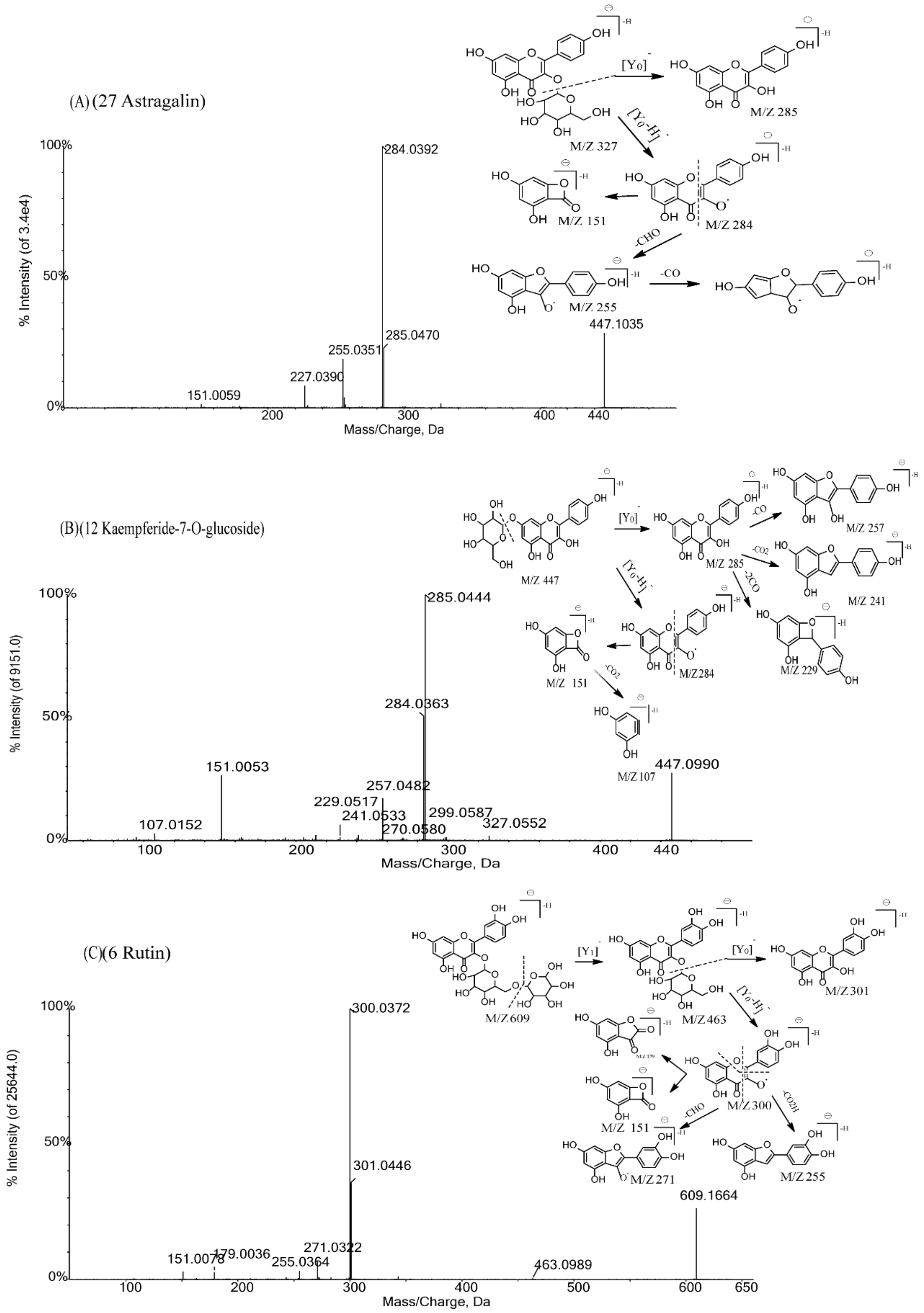

Fig. S2 The typical MS/MS spectrum of flavonoid-O-glycosides and its fragmentation pathway: (A) Astragalin, (B) Kaempferide-7-O-glucoside, (C) Rutin.

(A) (37 vitexin)

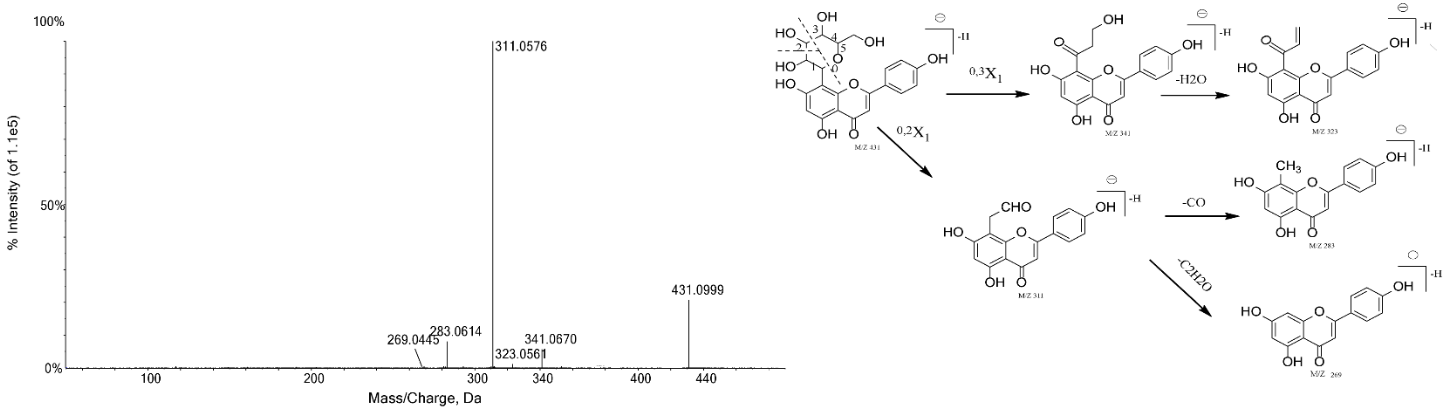

(B)(5 Schaftoside)

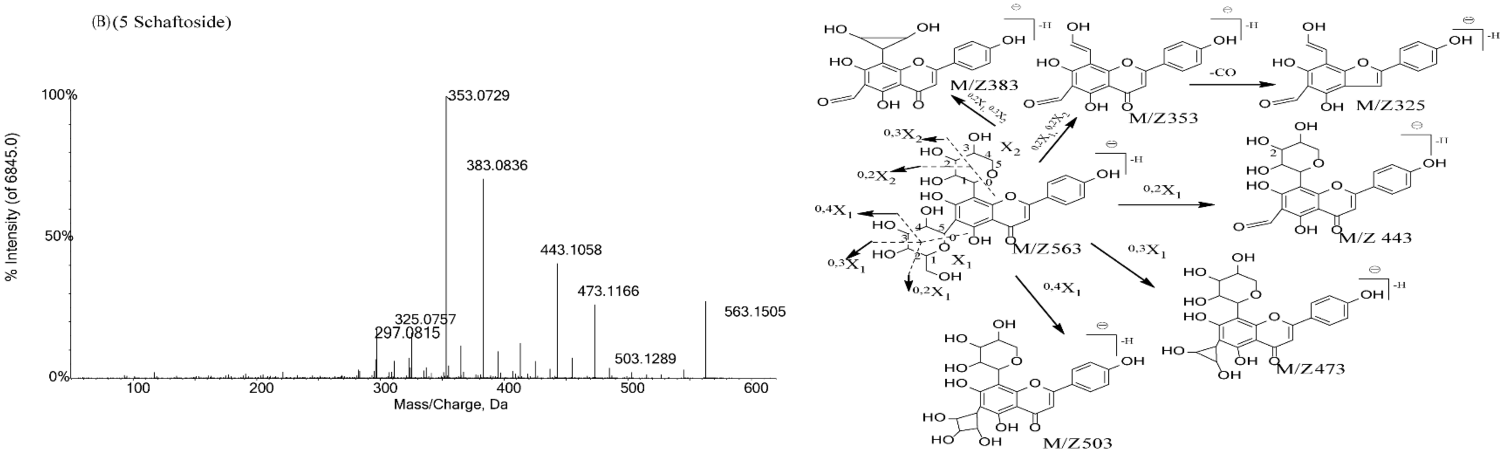

Fig.S3 The typical MS/MS spectrum of flavonoid-C-glycosides and its fragmentation pathway: (A) Vitexin, (B) Schaftosi.

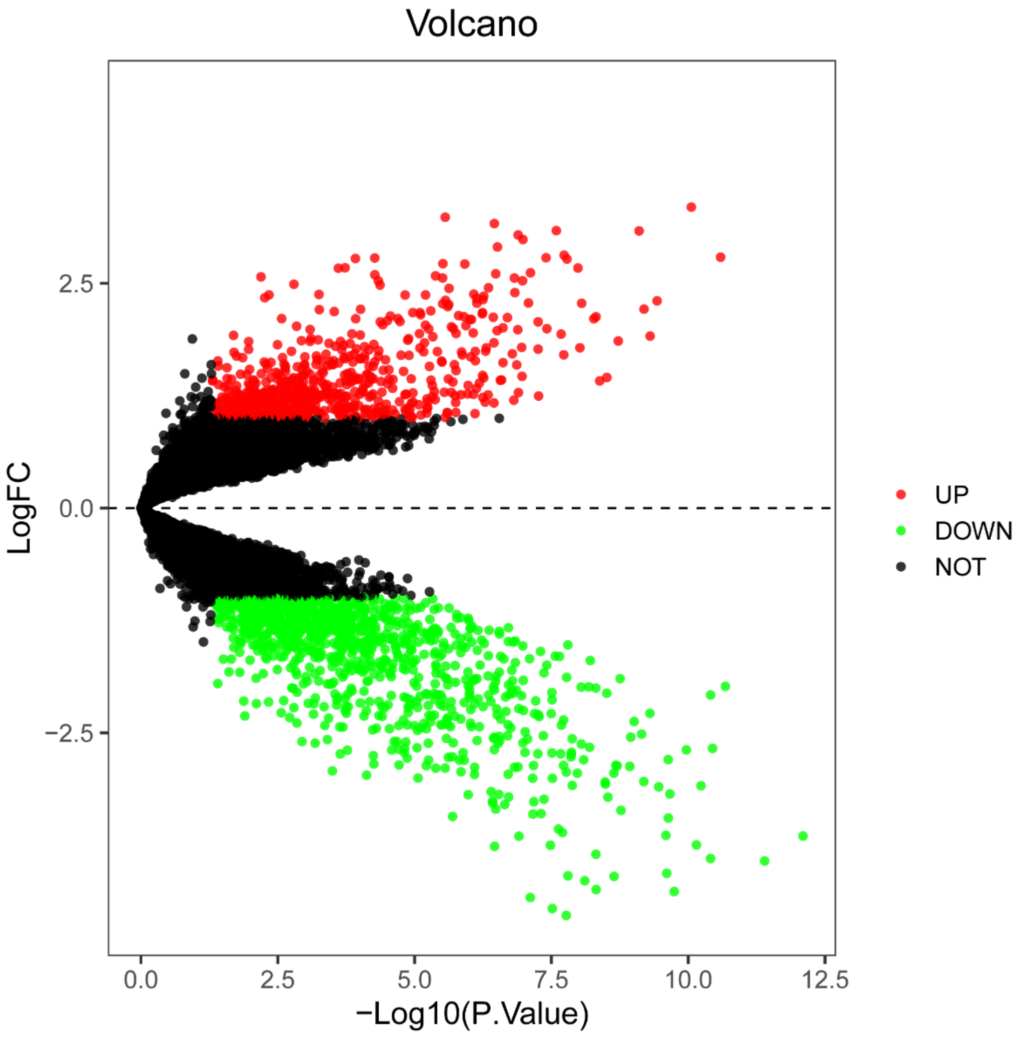

Fig.S4 The volcano map of the differential genes in healthy human liver tissue and liver cancer tissue.

Table S1 Window distributions of variable window SWATH methods.

Experiment	MS Type	Min m/z	Max m/z
0	SCAN	49.5	999.5
1	SWATH	49.5	69.5
2	SWATH	68.5	72.5
3	SWATH	71.5	86.5
4	SWATH	85.5	107.5
5	SWATH	106.5	115.5
6	SWATH	114.5	129.5
7	SWATH	128.5	136.5
8	SWATH	135.5	159.5
9	SWATH	158.5	173.5
10	SWATH	172.5	180.5
11	SWATH	179.5	191.5
12	SWATH	190.5	241.5
13	SWATH	240.5	279.5
14	SWATH	278.5	285.5
15	SWATH	284.5	287.5
16	SWATH	286.5	353.5
17	SWATH	352.5	474.5
18	SWATH	473.5	490.5
19	SWATH	489.5	592.5
20	SWATH	591.5	999.5

Table S2 Classification results of 16 unknown samples.

Name	Probability	Class
Unknown-1-Taiju	0.93333	Taiju
Unknown-2-Taiju	0.99667	Taiju
Unknown-3-Taiju	0.99333	Taiju
Unknown-4-Taiju	0.99333	Taiju
Unknown-5-Taiju	0.93333	Taiju
Unknown-6-Taiju	0.99667	Taiju
Unknown-7-Taiju	0.99333	Taiju
Unknown-8-Taiju	0.99333	Taiju
Unknown-9-Duoju	0.86667	Duoju
Unknown-10-Duoju	0.95667	Duoju
Unknown-11-Duoju	0.93333	Duoju
Unknown-12-Duoju	0.92667	Duoju
Unknown-13-Duoju	0.86667	Duoju
Unknown-14-Duoju	0.95667	Duoju
Unknown-15-Duoju	0.93333	Duoju
Unknown-16-Duoju	0.92667	Duoju

TableS3 Results of KEGG pathway information.

GO	Description	Hits	Log(q-value)
hsa04914	Progesterone-mediated oocyte maturation	CCNA2|CDK1|CDC25A|PIK3R1|PLK1|CCNB3	-10.0365
hsa04110	Cell cycle	CCNA2|CDK1|CDC25A|PLK1|CCNB3	-7.3006
hsa04218	Cellular senescence	CCNA2|CDK1|CDC25A|PIK3R1|CCNB3	-7.00795
hsa05203	Viral carcinogenesis	CCNA2|CDK1|PIK3R1	-2.76769
hsa05170	Human immunodeficiency virus 1 infection	CDK1|PIK3R1|CCNB3	-2.76769
hsa04068	FoxO signaling pathway	PIK3R1|PLK1|CCNB3	-3.18848
hsa04114	Oocyte meiosis	AR|CDK1|PLK1	-3.18848
hsa05207	Chemical carcinogenesis - receptor activation	AR|CDC25A|PIK3R1	-2.76769
hsa05200	Pathways in cancer	AR|CCNA2|PIK3R1	-1.64308
